# Gingival health in relation to clinical crown length: a case report

**DOI:** 10.1186/1757-1626-2-9387

**Published:** 2009-12-23

**Authors:** Alf Volchansky, Peter Cleaton-Jones

**Affiliations:** 1 Department of Experimental Odontology and Orthodontics, School of Oral Health Sciences, University of the Witwatersrand, Johannesburg, 2050 Wits, South Africa; 2 Department of Maxillofacial and Oral Surgery, School of Clinical Medicine, University of the Witwatersrand, Johannesburg, 2050 Wits, South Africa

## Abstract

**Introduction:**

Gingival margin position in relation to synthetic crowns and crown length could be etiological factors in gingival health.

**Case presentation:**

A 27-year-old male presented with necrotizing ulcerative gingivitis with short clinical crowns suggestive of altered passive eruption. Three years after the initial diagnosis, he presented with crowns on the maxillary incisors. There were short clinical crowns and marked gingival inflammation.

**Conclusion:**

Placement of the crown margin could be an etiological factor in gingival inflammation. Therefore, should the margin be subgingival, equigingival or supragingival?

## Case presentation

A 27-year-old white South African male presented at a periodontal practice complaining of painful "gums" particularly in the anterior part of the mouth, and halitosis. His general health was satisfactory; he was a non-smoker and other than grinding his teeth was not aware of any etiological factors, such as mouth breathing, that could have contributed to his problems.

After examination a diagnosis of necrotizing ulcerative gingivitis (NUG) was made, based on the classical punched out papillae, combined with the patient's symptoms of pain and halitosis.

**Figure 1:**
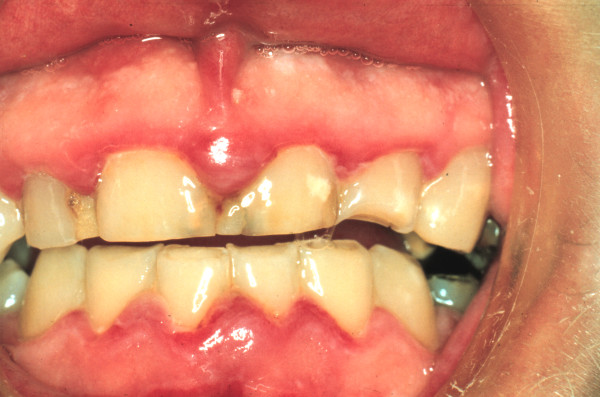
**Clinical photograph of the anterior gingiva of a 27-year-old male showing *necrotizing ulcerative gingivitis *(NUG)**.

The clinical picture was, however, more complex. In addition to the classical punched out papillae seen with NUG, there was inflammatory gingival hyperplasia, and marked occlusal wear (probably due to his grinding habit), and short clinical crowns, all of which, in the presence of plaque could contribute to the gingival inflammation (Figure 1).

**Figure 2:**
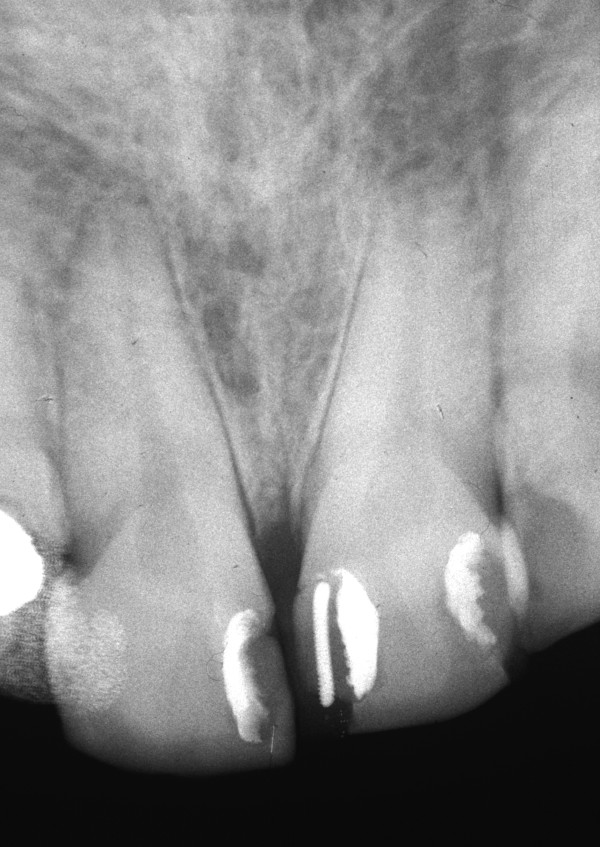
**Intra-oral radiograph of the maxillary incisors at time of NUG diagnosis**. The angle of the x-ray has shortened the roots.

An intra-oral radiograph (Figure. 2) showed that the incisor teeth were not fully erupted. While the short clinical crowns could be due to occlusal wear or delayed tooth eruption, another possibility was that *altered passive eruption *(APE) was present, which is a predisposing factor to NUG [1]. Metronidazole 200 mg tablets three times a day were prescribed at the first visit and combined with palliative treatment, consisting of scaling, home care instruction and a mouthwash.

**Figure 3:**
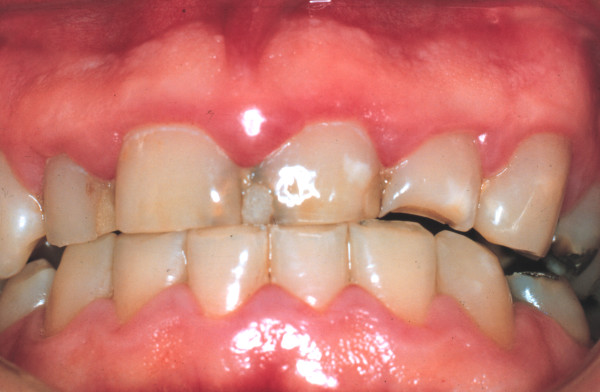
**Clinical photograph of the anterior gingiva after palliative periodontal treatment**.

**Figure 4:**
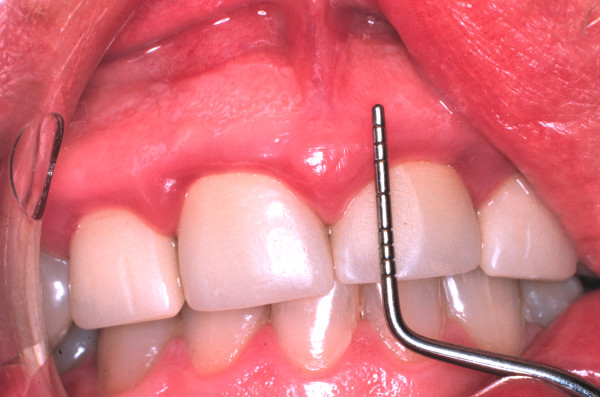
**Clinical photograph showing synthetic crowns and a periodontal probe indicating clinical crown length**.

**Figure 5:**
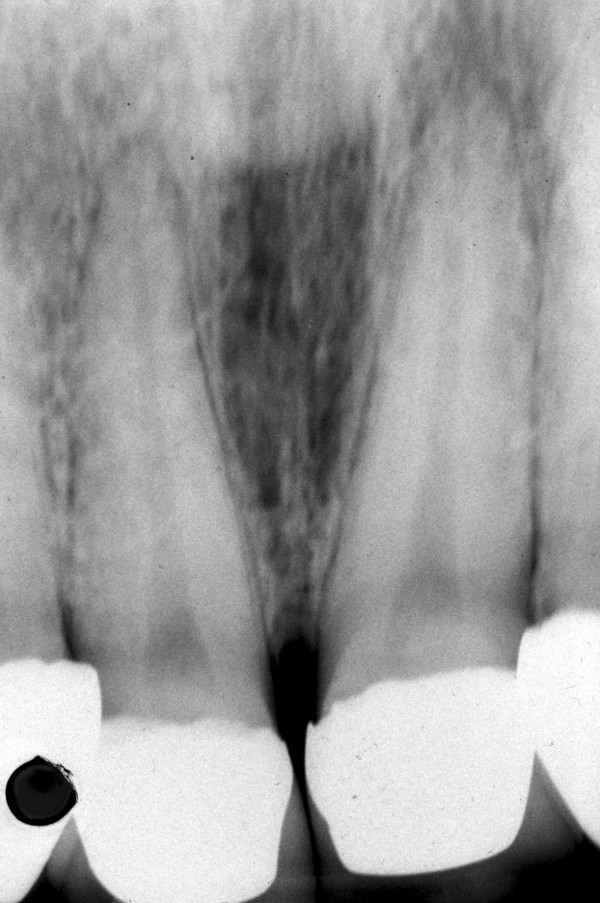
**Intra-oral radiograph indicating the crown margins in relation to the cemento-enamel junction**.

One week later, there was some resolution of the gingival inflammation (Figure 3). Possible gingival and periodontal surgical correction of the apparent gingival overgrowth was discussed with the patient who chose not to have this treatment. Thereafter he was seen intermittently; three years after the initial consultation he presented with synthetic crowns on the maxillary incisors, placed there by another practitioner. The associated gingivae were red, shiny with rolled margins and marked inflammation (Figure 4). Clinical examination also showed the presence of APE which we have defined as "when a tooth has reached the occlusal plane and the gingival margin in the mid-line of the tooth is at the junction between the cervical and middle third of the clinical crown; or in the middle third or coronal third of the clinical crown in the absence of inflammation, hypertrophy or hyperplasia of the gingiva" [2]. A periodontal probe showed clinical crown length (Figure 4) and an intra-oral radiograph (Figure 5) confirmed, that the crowns were not placed at or adjacent to the cemento-enamel junction (CEJ), but markedly coronal to it, as one would expect if APE is not recognized.

The synthetic crown margins were just apical to the gingival margin, a long way from the CEJ, on the convex facial surface of the clinical crowns, in a position conducive to trauma from "food impaction" and the accumulation of plaque, contributing to chronic inflammation [3].

## Discussion

Synthetic crown margin placement, clinical crown size/length, crown contour and biological width are important etiological factors in gingival and periodontal health.

Many years ago, a clinician had three options for crown margin placement; it could be supragingival, equigingival or subgingival [4]. Newcomb [5] indicated that subgingival margins were associated with plaque accumulation and gingival inflammation. Twenty years later, Sorensen [6] stated that subgingival margins greatly increase the frequency of periodontal disease, and that surface roughness, marginal fit and crown contour, mediate plaque accumulation and influence gingival health. This has also been reported for posterior crowns where bleeding was greater with sub-gingival crown placement [7].

Esthetics versus health is also a consideration [8] since subgingival finish lines are not periodontally advantageous, although they are required in certain situations. Currently, new materials and restorations may be finished easily to provide a smooth, polished interface at the gingival margin, so that plaque accumulation may be less [9,10].

There is more concern about the impingement of the *biologic width *[11,12] which is the physiological dimension of the junctional epithelium and connective tissue attachment, also described as the combined connective tissue - epithelial attachment from the crest of the alveolar bone to the base of the gingival sulcus. There is a view that gingival inflammation is influenced by clinical crown size and the pseudo pocket of APE [13].

Spear and Coonen [9] described a patient with a short clinical crown having an altered eruptive pattern and a sulcus depth of more than 3 mm. In such an instance a clinician must evaluate if a gingivectomy could be performed to lengthen the teeth and create a 1.5 mm sulcus.

As with coronally placed gingival margins the facial and lingual enamel bulges (crown contour) of human teeth protect the free gingival margin from the trauma of occlusion by deflecting food over the gingival crevice and onto keratinized gingival tissue [14,15].

## Conclusion

Even with new technology the position of a synthetic crown is crucial to gingival health. Therefore, is it not time to revisit the questions?

• should the crown margin be subgingival, equigingival or supragingival?

• how significant is clinical crown length and contour?

• is the biological width sacrosanct?

## Consent

The patient left the country and did not return to follow-up. Both authors agreed to publication on an anonymous basis.

## Competing interests

The authors declare that they have no competing interests.

## Authors' contributions

AV treated the patient. Both authors shared the writing of the manuscript, both read and approved the final manuscript.
